# Malignant Pheochromocytoma with Widespread Bony and Pulmonary Metastases

**DOI:** 10.7759/cureus.3348

**Published:** 2018-09-24

**Authors:** Tazeen Muneer, Aisha Tariq, Asif H Siddiqui, Muneer Amanullah

**Affiliations:** 1 Emergency Medicine, Glangwili General Hospital, Carmarthen, GBR; 2 Miscellaneous, The Aga Khan University Hospital, Karachi, PAK; 3 Surgery, The Aga Khan University Hospital, Karachi, PAK; 4 Cardiothoracic Surgery, The Aga Khan University Hospital, Karachi, PAK

**Keywords:** metastatic pheochromocytoma, malignant pheochromocytoma, pheochromocytoma

## Abstract

Pheochromocytoma is a rare benign tumor of the adrenal gland. A select few cases may be malignant, and metastatic cases are exceedingly rare. It often presents with symptoms of catecholamine excess, such as sweating, palpitations, headaches, and characteristic paroxysmal hypertension. Due to its diffuse symptoms, it is difficult to diagnose and is often diagnosed late. We describe the unique case of a 44-year-old female patient who presented with uncontrolled hypertension and vomiting, accompanied by lower back pain. She was diagnosed with malignant pheochromocytoma with multiple metastases to the lungs, vertebrae, scapulae, and skull. Because of the advanced state of her disease, the patient was started on treatment with the chemotherapeutic combination of cyclophosphamide, vincristine, and dacarbazine. However, she had a complicated hospital course and died because of aspiration pneumonia and sepsis.

## Introduction

Pheochromocytomas are tumors of chromaffin cells present in adrenal glands. In 1905, Poll described the tumor as a dusky (pheo) colored (chromo) tumor (cytoma), and hence brought about its name ‘pheochromocytoma’ [1]. While these tumors can present with only compressive symptoms, they are notorious for being a source of excess catecholamines such as epinephrine and norepinephrine. This can result in signs and symptoms such as excessive sweating, headaches, palpitations, dizziness, and characteristic paroxysms of hypertension, where blood pressure shows volatility induced by stressful situations [1-2]. Owing to the diversity of clinical signs and symptoms with which the tumor presents, often, the tumor is either diagnosed late or is missed and diagnosed post-mortem. Here, we report a case of malignant pheochromocytoma with distant metastases to the vertebral bodies.

## Case presentation

A 44-year-old lady presented to the emergency department with vomiting for four days and uncontrolled hypertension. Blood pressure was poorly controlled (230/130 mmHg) due to the erratic use of antihypertensives over the past 25 years. She also had lower back pain, managed by non-steroidal anti-inflammatory drugs (NSAIDs). Her review of systems was only significant for chronic renal insufficiency and a past history of liver abscess.

On examination, she was afebrile but hypertensive, with a blood pressure of 180/100 mmHg and a pulse of 160/min. She was alert and oriented to time, place, and person. The respiratory and cardiovascular examination yielded no findings; however, on abdominal examination, there was right upper quadrant tenderness on palpation. Her liver and spleen were not palpable.

Lab investigations of the patient were as shown in Table 1. She had an abnormal white blood cell count with neutrophilia and elevated platelet count, creatinine, blood urea nitrogen (BUN), calcium, and phosphate levels. She also had abnormal liver function tests.

**Table 1:** Laboratory investigations (blood) All normal ranges are quoted from the Aga Khan University Hospital Laboratory. Key: BUN, blood urea nitrogen

Test (unit)	Results	Normal range
White blood cell count (cells/L)	17.9	4.0 – 10.0
Neutrophils (%)	82.1	40 – 75
Lymphocytes (%)	11.1	20 – 45
Platelet count (mg/dL)	517, 000	150 – 400
Creatinine (mg/dL)	2.3	0.65 – 1.1
BUN (mg/dL)	42	4 – 15
Calcium (mg/dL)	10.8	8.6 – 10.5
Albumin (g/dL)	3	3.2 – 5
Phosphate (mg/dL)	8.1	2.7 – 4.8
Aspartate transaminase (IU/L)	56	18 – 32
Alanine transaminase (IU/L)	27	3 – 33
Gamma glutamyl transferase (IU/L)	49	1 – 37
Alkaline phosphatase (IU/L)	211	29 – 132

**Figure 1:**
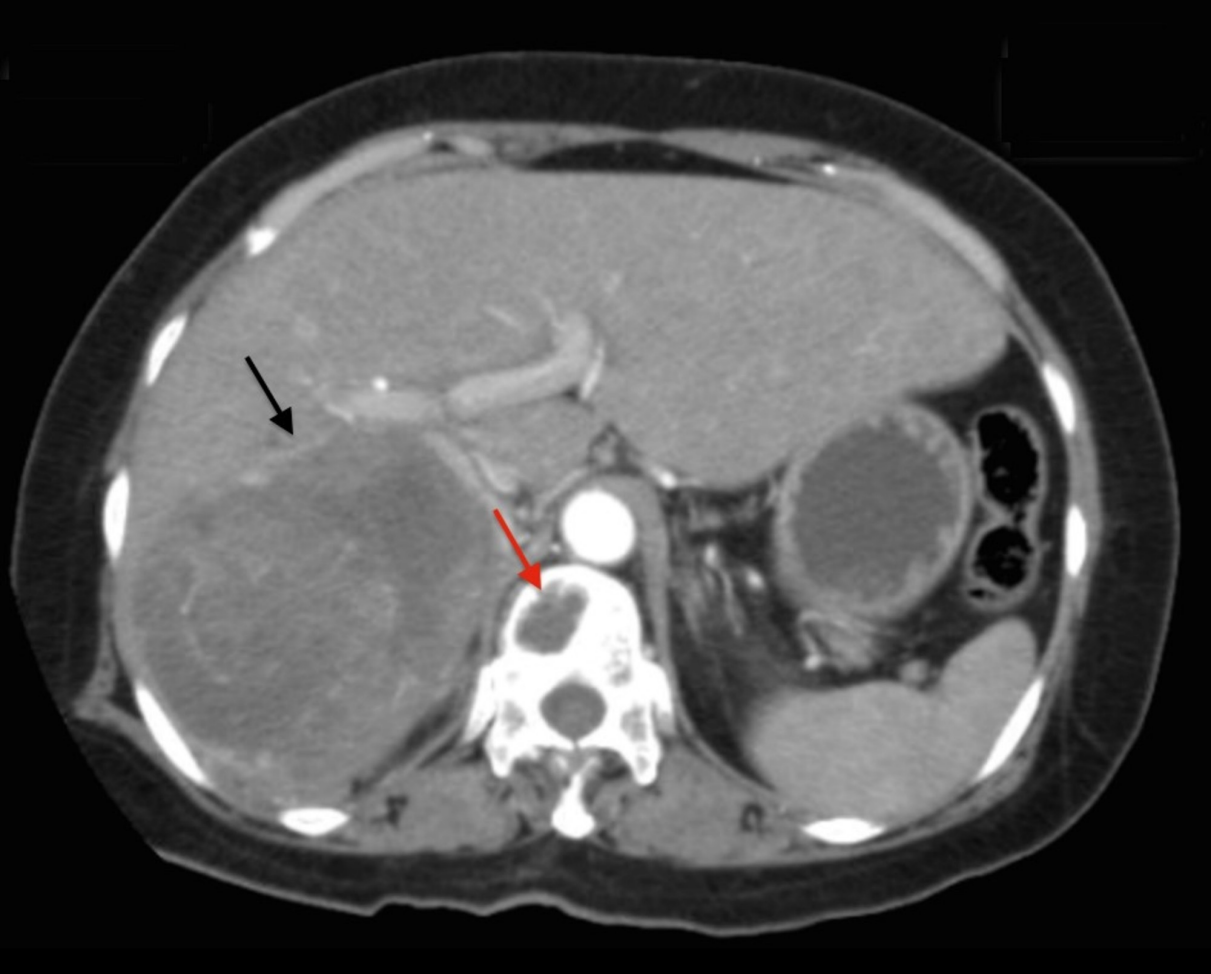
Axial section at the level of T12 from the CT scan of the patient showing the adrenal mass (black arrow) and a lytic lesion in the vertebra (red arrow) CT: computed tomography

**Figure 2:**
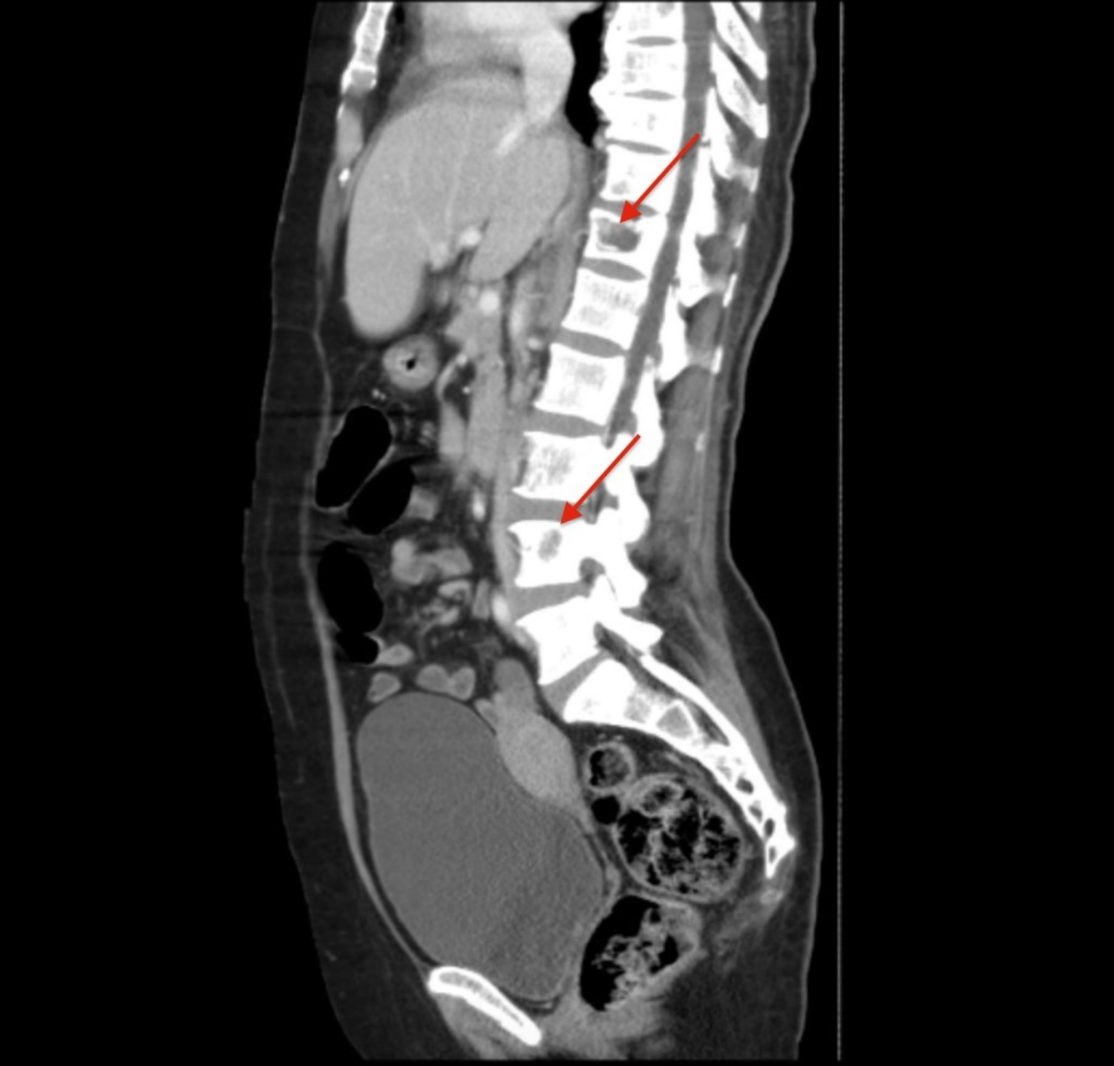
Sagittal section of a CT scan showing multiple lytic lesions in the thoracic spine and lumbar spine (red arrows) CT: computed tomography

**Figure 3:**
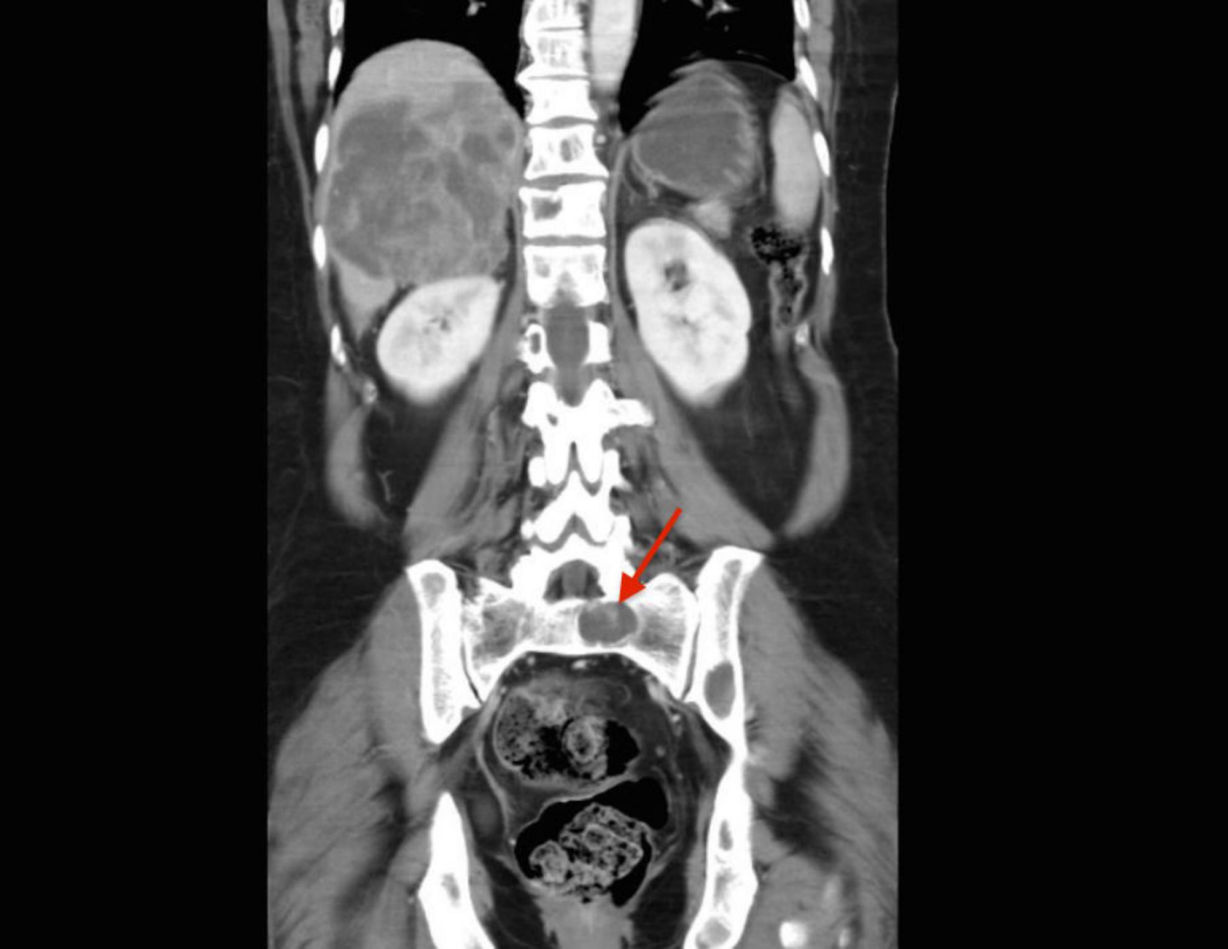
Coronal section of the patient's CT scan showing a lytic lesion in the left sacrum of the patient (red arrow) CT: computed tomography

**Figure 4:**
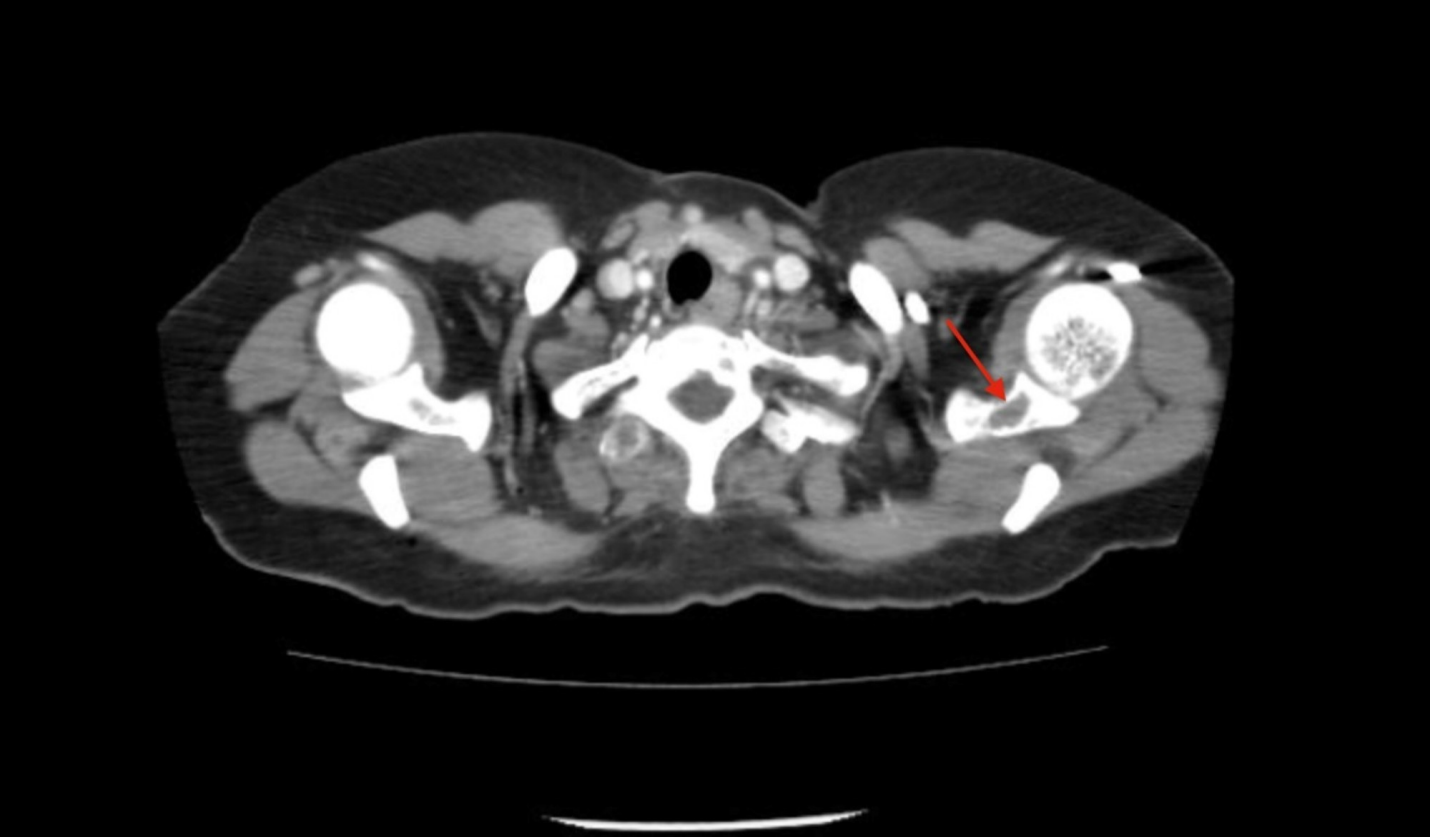
Axial section of a CT scan showing a lytic lesion in the left scapula (red arrow) CT: computed tomography

**Figure 5:**
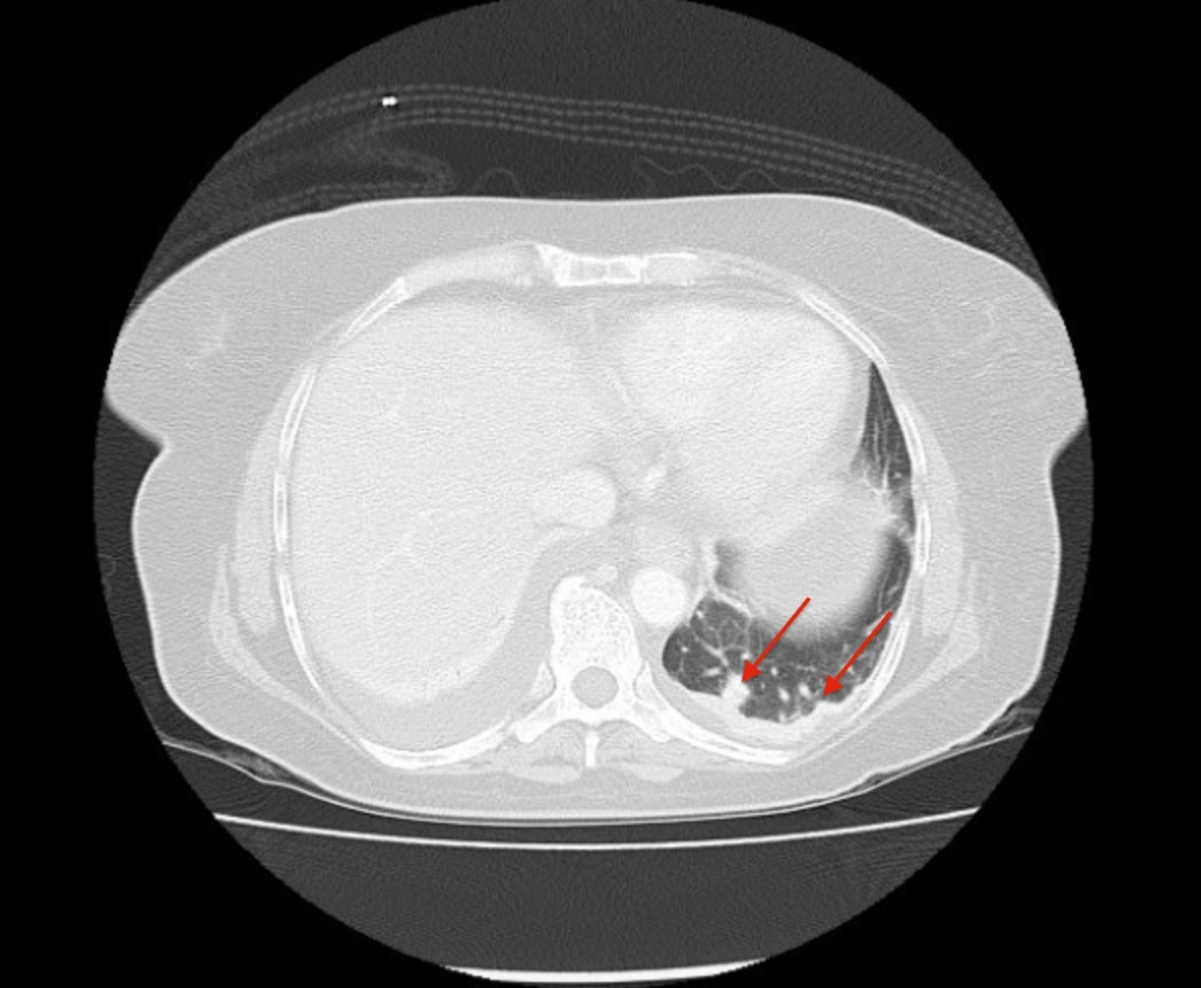
Axial section of the patient's CT scan showing nodules in the lower left lung (red arrows) CT: computed tomography

The abdominal ultrasound revealed a 10.3 x 9.6 x 6.7 cm heterogeneous cystic lesion in the right lobe of the liver. A computed tomography (CT) scan of the chest and abdomen showed that the suspected liver mass was actually an 11.3 x 8.0 cm heterogeneous mass in the right adrenal gland with a normal liver and spleen (Figure 1). There were no signs of lymphadenopathy. Lytic lesions were noted in the left acetabulum, sacrum, right and left iliac blades, and the lumbosacral and thoracic spine (Figures 2-3). The chest showed two nodules in the left lung and multiple lytic lesions in the scapula and multiple vertebrae (Figures 4-5).

Ovarian and gastrointestinal pathologies were ruled out; however, lactate dehydrogenase (LDH) was elevated (1080 IU/L). The patient was tested for 24-hour urine vanillylmandelic acid (VMA), which was significantly high at 175 mg (normal < 13.6 mg). She was investigated for the possibility of endocrine syndromes, such as multiple endocrine neoplasias (MEN) I or II; however, her parathyroid hormone levels were within normal limits at 37.30 pg/mL and an ultrasound of her thyroid gland showed no focal lesions. Thyroid stimulating hormone (TSH) and calcitonin were within normal limits.

Her blood pressure was controlled using α- and β-blockers. A biopsy of the left acetabular lesion was positive for cytokeratin AE1/AE3, chromogranin A, and neuron-specific enolase (NSE) antibodies, consistent with the diagnosis of metastatic pheochromocytoma. Due to the extent of her disease, excision of her primary lesion was not done, and she was started on chemotherapy with cyclophosphamide, vincristine, and dacarbazine. Her clinical course was complicated with febrile neutropenic episodes, and she died three months later, secondary to aspiration pneumonia and sepsis.

## Discussion

Pheochromocytomas are tumors of chromaffin cells, originating embryonically from neuroendocrine cells. Chromaffin cells are found concentrated in the adrenal gland, which explains why pheochromocytomas occur there most frequently [1]. About 90% of all pheochromocytomas are found in the adrenal gland and rarely occur above the diaphragm. Paragangliomas (a name for pheochromocytomas present outside the adrenal gland) are usually found intra-abdominally, along the sympathetic chain, or in the organs of Zuckerkandl. Less than one percent of them are found in the thoracic cavity, in rare sites such as the heart and the mediastinum [1]. Due to their unique nature of secreting catecholamines, these tumors can present with the characteristic sign of paroxysmal hypertension. When these catecholamines are metabolized, they form metanephrines and VMA, which are excreted via the kidneys and are used for diagnosis [3].

Pheochromocytomas are also associated with other endocrine tumors, as seen in syndromes such as MEN I, MEN II, von Hippel Lindau disease, and neurofibromatosis type 1. They can present with pancreatic tumors, parathyroid hyperplasia, and/or thyroid cancer. It has also been linked to mutations in the gene for succinate dehydrogenase (SDHD) subunits, rearranged during transfection (RET), and many other genes, which are autosomal dominant in nature [4].

The incidence of pheochromocytomas is 2-8 per million people. Of these cases, about 10%-15% turn out to be malignant and only a further 10% metastasize [3-4]. Observing capsular and/or vascular invasion and the presence of distant metastases identifies malignant pheochromocytomas. According to a study by Thompson et al. [2], other features used to ascertain malignant potential are the presence of necrosis, the presence of nests/diffuse growth, high cellularity, tumor cell spindling, increased mitotic figures, and profound nuclear pleomorphism. While pheochromocytomas most commonly present with paroxysmal hypertension, most malignant pheochromocytomas are diagnosed when distant metastases have occurred, usually to regional lymph nodes, bones, brain, liver, and lungs [5]. Often, patients can present with bone pains and fractures due to bone metastases. Yamaguchi et al. [6] reported a patient who initially presented with a C4 fracture and was being treated with collar fixation. Similarly, a patient with a history of a carotid body tumor presented five years after the successful resection of her tumor with a pathologic fracture of C5, T5 and T11 [7].

Due to the rarity of this tumor, there is limited data available regarding the presentation and management of malignant pheochromocytomas. While some centers practice surgical interventions, such as open laparotomy and regional lymphadenopathy for local disease, studies are now looking into chemotherapy and radiotherapy as alternatives [8]. According to the National Comprehensive Cancer Care (NCCN) guidelines, while all tumors are initially managed conservatively with salt restriction, beta blockers, alpha blockers, and/or calcium channel blockers, definitive treatment varies for surgically resectable, unresectable, and metastatic tumors. Surgically resectable tumors can be removed preferentially via laparoscopy whereas unresectable tumors can require radiotherapy, iodine-131 metaiodobenzylguanidine (131I-MIBG) therapy, which is a norepinephrine analog, and/or peptide receptor radionuclide therapy (PRRT) with Lu-dotatate, for somatostatin receptor-positive tumors [9-10]. Symptomatic metastatic tumors can be managed with systemic chemotherapy such as the combination of cyclophosphamide, vincristine, and dacarbazine (CVD). CVD has been reported to have a 51% survival rate among patients with malignant pheochromocytomas. Thirty-three percent of these patients showed a clinical and radiological response to CVD [11]. Other options include 131I-MIBG, Lu-dotatate, and/or radiotherapy.

Sutinib, a tyrosine kinase inhibitor, was approved in 2006 to treat renal cell carcinoma and neuroendocrine tumors. It works by reducing hypoxia-induced factor (HIF) transcription factors, which have been associated with increased cell growth and angiogenesis. They are present in high concentrations in cells with germline mutations, such as VHL and SDHD; mutations that are heavily associated with pheochromocytomas [12].

## Conclusions

Regardless of advances being made in forming innovative and effective treatments for pheochromocytoma, the prognosis remains poor, as most cases are identified after metastasis has occurred and the disease has advanced significantly. Malignant pheochromocytoma remains a rare tumor that has a complicated and difficult road to recovery. We recommend the publication of more data in the form of case reports and case series along with the treatments employed at centers worldwide so that a standardized evidence-based protocol can be devised for the management of patients with this condition and the prevention of complications.
